# Effects of Coworkers’ Helping Behavior on Employees’ Knowledge Sharing and Creativity: The Moderating Role of Interactional Justice

**DOI:** 10.3390/ijerph182413302

**Published:** 2021-12-17

**Authors:** Soojin Lee, Gukdo Byun, Suzi Kim

**Affiliations:** 1 College of Business Administration, Chonnam National University, Gwangju 61186, Korea; soojinlee@jnu.ac.kr; 2 College of Business, Chungbuk National University, Cheongju 28644, Korea

**Keywords:** coworkers’ helping behavior, knowledge sharing, creativity, interactional justice

## Abstract

Although it is important to examine how creative performance can be achieved by facilitating knowledge sharing activities among its members, few studies have examined these relationships. Therefore, this study analyzed the effects of coworkers’ helping behavior on knowledge sharing and creativity. It also attempted to demonstrate the moderating role of interactional justice as a situational variable that positively affects coworkers’ helping behavior. Using data from 200 full-time supervisor–subordinate dyads in a large public institution located in South Korea, we performed multiple regression analysis and the bootstrapping method to test our hypotheses. The results of this study presented that coworkers’ helping behavior encouraged individuals to share knowledge and increase their creative performance. Moreover, this study demonstrated that the positive effect of coworkers’ helping behavior on employees’ creativity through their knowledge sharing was stronger when interactional justice was high rather than low. Therefore, this research contributes to finding the critical factors that enable a company to gain a competitive advantage by providing the impact of coworkers’ helping behavior and supervisors’ interactional justice on knowledge sharing and creativity among employees.

## 1. Introduction

Rapid technological developments and a changing global order have rendered securing a sustainable competitive advantage essential for the survival of an organization [1]. A critical contributor to an organization’s competitive advantage is employee creativity [2,3], based on which it seeks to make important changes and innovations [4]. The creative performance of employees is a strategy to ensure an organization’s innovative behavior, productivity, and survival [5]. Creative performance is generated and expanded by sharing and re-integrating employees’ knowledge [6]. Therefore, to increase creative performance, it is important that the members of an organization share their knowledge and reinforce the value of such knowledge [7]. Some researchers have suggested that knowledge sharing is deeply related to innovative behavior and plays an important role in improving organizational competitiveness [8].

The role of organizational members is critical in promoting knowledge sharing and creativity. In an organization, employees form business and psychological relationships while performing tasks. Specifically, interactions among coworkers occur frequently in teamwork, which is the norm these days. In such an environment, it is essential to help each other and cooperate with coworkers while performing tasks [9]. A helping behavior can benefit both the individuals receiving help and the organization they belong to as it provides ideas and assistance to share and solve problems [10]. Hence, examining how voluntary and cooperative behavior among employees can lead to outcomes that help an organization gain a competitive advantage, such as knowledge sharing and creative performance, is an important research topic. As such, this study sought to analyze how coworkers’ helping behavior, as a voluntary and cooperative variable, influences employees’ knowledge sharing and creativity.

In addition, from a social exchange theory perspective, employees contribute to their organization or engage in voluntary behavior based on positive expectations from and trust in those with whom they have a social exchange. In an organization, employees engage in social exchanges with their supervisors and coworkers [9]. Accordingly, employees’ perception of their supervisors serves as a key situational factor in determining their behavior. In other words, even if employees receive a lot of help from their coworkers, they may perceive injustice if they feel alienated from personal relationships with their supervisor or believe that information is shared unequally. It may significantly undermine the positive ripple effects of coworkers’ helping behavior. In contrast, if employees perceive interactional justice from their supervisor, it increases their sense of belonging and cooperative behavior [11] and reinforces their inherent motivation, thus resulting in greater knowledge sharing and creativity because of coworkers’ helping behavior [12]. Considering that supervisors and coworkers within the organization are important interaction targets for employees [9], the perception of interactional justice can play a critical role in determining their knowledge sharing through the helping behavior of their colleagues. Therefore, this study attempts to demonstrate the moderating effect of supervisors’ interactional justice on the relationship between coworkers’ helping behavior and employees’ knowledge sharing and creativity.

The purposes of this study are summarized as follows. First, this study aims to identify the relationship between coworkers’ helping behavior and employees’ knowledge sharing. The most important motive for knowledge sharing is an individual’s willingness to share knowledge. Therefore, this study demonstrates that the behaviors to help colleagues and alleviate their difficulties can lead to knowledge sharing, which is voluntary and discretionary behavior on the part of employees. Second, this study analyzes the positive effects of coworkers’ helping behavior as a way to improve creative performance. Voluntarily helping a colleague in difficulty can help the recipient look at the problem from a fresh perspective and increase the possibility of solving it in a new and creative way. Based on previous research, which reported that employee creativity increased when information or feedback was exchanged among coworkers [13], this study demonstrates the positive relationship between coworkers’ helping behavior and employee creativity. Finally, it examines the moderating effect of supervisors’ interactional justice on the relationship between coworkers’ helping behavior and employees’ knowledge sharing and creativity. By testing the moderating effect of interactional justice, this study determines whether a supervisor’s respect, consideration, and fair treatment can contribute to a positive perception of the organization and enhance the positive ripple effects that potentially arise from coworkers’ helping behavior.

Accordingly, this study is designed to identify the key factors that enable an organization to obtain a competitive advantage by demonstrating the effect of coworkers’ helping behavior and supervisors’ interactional justice on employees’ knowledge sharing and creativity.

## 2. Theoretical Background and Hypothesis Development

### 2.1. Coworkers’ Helping Behavior

Helping behavior refers to the voluntary performance of actions that go beyond those formally required to perform a task [14]. A good example is helping a coworker with a high workload or providing information on behalf of a coworker if they are absent. Van Dyne, Cummings, and Parks ([15], p. 218) defined extra-role behavior as “behavior which benefits the organization and/or is intended to benefit the organization, which is discretionary and which goes beyond existing role expectations”. The importance of cooperative and voluntary helping behavior among employees is increasingly emphasized as actions to help coworkers outside of delineated role expectations, which are considered valuable and beneficial to the organization [16].

While extra-role behavior is not officially stated in the description of task activities, employees form social relationships with coworkers by providing and receiving help as organization members. In particular, in today’s organizational system where employees commonly work in teams, mutual exchange of help among colleagues plays a key role in improving performance and establishing a culture in the organization. Specifically, coworkers’ helping behavior increases employees’ personal resources and leads to their positive attitude and performance [17]. Furthermore, those receiving help can preserve and develop resources in an organization through relationships in which they acquire these resources [18].

### 2.2. Coworkers’ Helping Behavior and Knowledge Sharing

Knowledge is valuable information a person possesses, and it has the characteristics of being implicit and difficult to structure and transfer [19]. Knowledge sharing can be defined as the holistic process by which employees explore, disseminate, and acquire comprehensive knowledge, which encompasses explicit and implicit knowledge, through interactions with each other [20]. Thus, knowledge sharing refers to an activity that seeks to maximize the use of knowledge and improve organizational competencies by sharing the knowledge or assets in an organization [21].

Factors that improve or undermine knowledge sharing have been studied from various internal and external environmental perspectives [22]. Wang and Noe [23] categorized the factors that affect knowledge sharing as structural, relational, and cultural. To highlight the importance of knowledge creation in an organization, the importance of knowledge sharing has been explained from a resource-based view. An organization’s growth or competitiveness is determined not by the external environment but its internal resources [24]. The knowledge held by an organization is also highly valuable as a resource. Therefore, many attempts have been made to determine ways to increase knowledge sharing, since sharing and using an organization’s knowledge to maximize its value is an important factor in improving its competitiveness. Hargadon [25] argued that an individual’s willingness to share knowledge with others is one of the most important factors in increasing knowledge sharing. Accordingly, if coworkers, who may otherwise become competitors in an organization, voluntarily and actively exhibit helping behavior, employees will be more inclined to share knowledge, which is a major asset they have, in the context of reciprocity.

Knowledge sharing is a type of extra-role behavior executed voluntarily for the benefit of other employees [26,27]. This aspect is aligned with helping behavior, or an altruistic and voluntary act. Therefore, coworkers’ helping behavior may act as an antecedent variable for knowledge sharing, considering that it can increase employees’ intention to provide help for the benefit of others rather than themselves. Employees who receive considerable help from coworkers may engage in more knowledge sharing activities based on the altruistic motivation that they should give back to others [28]. Based on a study finding that an individual’s intention to share knowledge comes from their willingness to do so, coworkers’ increased helping behavior is expected to increase employees’ altruistic motivation to give back and subsequently lead to more knowledge sharing. Based on the above discussion, the following hypothesis was established:

**Hypothesis** **1.** *Coworkers’ helping behavior has a positive relationship with employees’ knowledge sharing.*

### 2.3. Coworkers’ Helping Behavior and Creativity

Amabile [2] defined creativity as the ability to combine ideas in a unique way or connect them to relevant areas in a distinctive way. Creativity is expressed in processes, outcomes, and personal characteristics [2,29], and appears differently at the personal, group, organization, or society level, but it is mostly focused on the personal level [30]. Indeed, the findings of numerous empirical studies on the relationship between individual characteristics (e.g., big five personality, proactive personality, and emotional intelligence) and creativity have been proposed by many researchers [31,32,33,34]. However, there have been few empirical studies on the impact of coworkers’ helping behavior although social support has a significant impact on creativity.

Thus, this study suggests that coworkers’ helping behavior is a major antecedent factor for employees’ creativity. Altruistic behavior, such as helping others in difficulty in terms of work or in their absence, is considered to be part of a creative process where both those providing help and those receiving help participate. It is because coworkers’ helping behavior enables those receiving help to look at problems from various directions and solve them in creative ways. Studies have reported that creativity increases when coworkers exchange information or feedback, thereby suggesting that information exchange could have a significant effect on creativity [35]. In a study on helping behavior and creativity, Grodal, Nelson, and Siino [36] observed that the behavior of providing and receiving help could turn into a task pattern and lead to innovative behavior in an organization. Accordingly, through coworkers’ helping behavior, employees can increase their creative performance by collaborating with coworkers and sharing various problem-solving ideas. In other words, situations where employees receive a lot of help from coworkers are expected to enhance employees’ problem-solving skills and consequently their creative performance. Based on the above discussion, this study established the following hypothesis that coworkers’ helping behavior increases employee creativity.

**Hypothesis** **2.** *Coworkers’ helping behavior has a positive relationship with employees’ creativity.*

### 2.4. Mediating Effects of Employees’ Knowledge Sharing

Based on the arguments above, this study assumes that employees’ knowledge sharing mediates the relationship between coworkers’ helping behavior and employees’ creativity. As sharing knowledge with colleagues plays a fundamental role in the employee creative process [6], employees’ knowledge sharing behaviors are likely a key mechanism linking coworkers’ helping behavior with employees’ creative performance. As discussed earlier, when coworkers, who may be a potential competitor in an organization, exhibit voluntary helping behaviors to employees, these employees are more likely to share their knowledge and information accordingly. In addition, knowledge shared among organization members can be an important catalyst for individuals to achieve creative outcomes in the process of performing their duties.

Several studies have shown that members’ knowledge sharing enhances their creativity [6,37,38]. For example, according to the meta-analysis of Hülsheger et al. [39], the knowledge assembled within the team and the internal communication and sharing of this knowledge enable the team members to accomplish the complex tasks of developing new ideas and products. In addition, Dong et al. [6] suggested that knowledge sharing among team members leads to higher levels of team creativity and moderates the relationship between individual-focused transformational leadership, skill development, and individual creativity. They argued that the communication and sharing of individual knowledge in a team is a viable resource for the team to produce creative outcomes. In addition, Nonaka and Takeuchi [40] stated that knowledge sharing is essential to convert common ideas or thoughts into innovative outcomes. Based on the empirical evidence and discussions in previous research, we infer that employees’ knowledge sharing plays a key role in enhancing their creativity.

Consequently, combining the arguments underlying Hypotheses 1 and 2, we propose that coworkers’ helping behavior is positively related to employees’ knowledge sharing, which will then lead to higher levels of employee creativity. Thus, the following hypothesis was established:

**Hypothesis** **3.** *Employees’ knowledge sharing mediates the relationship between coworkers’ helping behavior and employees’ creativity.*

### 2.5. Moderating Role of Interactional Justice

Organizational justice refers to employees’ perceptions of fairness in their organization [41]. Organizational justice is an important variable in organizational behavior studies as it has a significant effect on employees’ attitude and task performance [42]. The concept of organizational justice has changed and evolved over time from equity theory to distributive justice, procedural justice, and interactional justice [43]. Bies and Moag [44] suggested the theory of interactional justice, which argues that employee perceptions of fairness depend on how they are treated in the process of decision-making or information communication. Thus, they defined interactional justice as employee perceptions of fairness in how they are treated by their supervisor in a procedure or process [44]. Furthermore, Tyler and Bies [45] suggested that interactional justice means behaviors that eliminate a leader’s personal bias, enable consistent decision-making, and facilitate the provision of timely feedback. As such, interactional justice is considered to be justice based on trust arising from the relationship with the supervisor, in contrast to distributive justice or procedural justice that arises at the organizational level [26].

Peer relationships within an organization are difficult to explain; however, the supervisor has the ability to significantly impact employees. Employees’ behavioral decisions are influenced by their leader [46], and the considerable effect of interactional justice on employees can be predicted. In fact, some researchers reported that employees engage in more organizational citizenship behavior if they perceive that they are treated fairly by their supervisor [47]. Based on these research results, it can be inferred that the higher the perceived level of interactional justice, the greater the positive effect of helping behavior, which is a type of organizational citizenship behavior. In other words, given that interactional justice has a positive effect on cooperative activities in an organization, it is possible to predict the moderating effect of interactional justice on the relationship between coworkers’ helping behavior and knowledge sharing and creativity.

Furthermore, Blau’s social exchange theory [48] suggests that employees feel obligated to give back when they feel they are treated fairly by their supervisor. If employees feel that their supervisor respects and supports them through interactional justice, their identity with the team is strengthened, and the possibility of cooperative behavior increases. In other words, when interactional justice is high, employees who receive help from coworkers feel it is fair to give back, which reinforces their intention to engage in reciprocal behavior. If the perceived level of interactional justice is high, employees who receive such help are also more willing to reciprocate and thereby contribute to their organization. Hence, more knowledge sharing is expected to occur.

In addition, a few researchers argued that individuals who perceive fairness can remain motivated to attain long-term goals, believing that their efforts can offset short-term losses with long-term gains [49]. Interactional justice can reduce the dilemma regarding short-term losses and keep employees motivated to realize an organization’s long-term goals as they feel their efforts would be properly recognized one day [49]. As knowledge sharing is also an exchange of value between employees, it is difficult to encourage it without creating an environment intended to recognize interactional justice in an organization. Thus, interactional justice can promote employees’ knowledge sharing, which is a positive effect of coworkers’ helping behavior.

Based on the above discussion, this study established the hypothesis that interactional justice strengthens the positive relationship between coworkers’ helping behavior and employees’ knowledge sharing.

**Hypothesis** **4.** *Interactional justice moderates the relationship between coworkers’ helping behavior and employees’ knowledge sharing, such that the relationship is stronger when interactional justice is high rather than low*.

Assuming that interactional justice moderates the relationship between coworkers’ helping behavior and employees’ knowledge sharing, it conditionally affects the strength of the indirect relationship between coworkers’ helping behavior and employees’ creativity. In other words, employees’ knowledge sharing conditionally influences the strength of the indirect effect of coworkers’ voluntary helping behavior on employees’ creative performance. This conditional effect is realized through the effect of interactional justice on employees’ creativity. Thus, the following hypothesis was established:

**Hypothesis** **5.** *Interactional justice moderates the positive and indirect effect of coworkers’ helping behavior on employees’ creativity through employees’ knowledge sharing. Specifically, the mediated relationship is stronger when interactional justice is high rather than low.*

**Figure 1:**
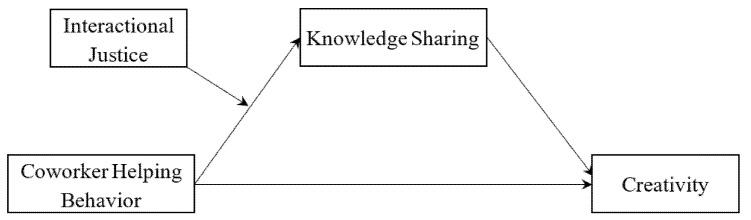
Hypothesized model.

Figure 1 presents the hypothesized model.

## 3. Methods

### 3.1. Sample and Procedure

To test the hypotheses, a questionnaire survey was distributed to full-time employees and their supervisors in a large public enterprise located in South Korea. This institution delivers numerous social security and labor welfare services and programs as a government-affiliated institute under the Ministry of Employment and Labor. Most of the participants work as clerical assistants on a regular basis. Questionnaires were distributed to 250 employees and their 250 immediate supervisors (leaders) in different places. After explaining the purpose of the study and how respondents’ personal information would be protected, the researchers distributed the questionnaire copies to the respondents and collected their responses. In total, 214 copies were collected from the employees and 216 copies from the supervisors, at a response rate of 85.6% and 86.4%, respectively. The final analysis used data from 200 pairs of the collected questionnaire copies after excluding copies with non-matched supervisor–employee pairs or incomplete responses, including missing values. The demographic characteristics of the sample indicated that the average age of employees was 33.47 years (SD = 4.32 years), 51% were men, and the average length of service with their leader was 12.45 years (SD = 13.34 years).

### 3.2. Measures

The traditional method of back translation [50] was used to translate the English language questionnaires into Korean. The questions were independently translated and back translated by two Korean bilingual academics. Validated scales from previous studies were used for all variables. To overcome common method bias, coworkers’ helping behavior and interactional justice were indicated by the employees, while creativity and knowledge sharing were evaluated by the supervisors. All questions were measured on a seven-point Likert scale (1 = strongly disagree; 7 = strongly agree).

#### 3.2.1. Coworkers’ Helping Behavior

From 14 organizational citizenship behavior scale items in Williams and Anderson [51], 7 items on organizational citizenship behavior focusing on individuals were used in the employee questionnaire to measure coworkers’ helping behavior. The items included the following: “My coworkers help others who have heavy work loads” and “My coworkers take time to listen to other employees’ problems and worries”.

#### 3.2.2. Knowledge Sharing

Knowledge sharing included seven items from Srivastava, Bartol, and Locke [22]. The supervisors were instructed to measure the level of employee knowledge sharing. The items included the following: “This employee exchanges information, knowledge, and sharing of skills with others” and “This employee offers many suggestions to others”.

#### 3.2.3. Creativity

The creative performance of employees was measured by the supervisors using 13 items from Zhou and George [13]. The items included the following: “This employee suggests new ways to achieve goals or objectives” and “This employee searches out new technologies, processes, techniques, and/or product ideas”.

#### 3.2.4. Interactional Justice

Interactional justice as perceived by the employees was measured using nine items in Colquitt [41]. Of the nine interactional justice items, four measure the supervisor’s interpersonal justice and five assess the supervisor’s informational justice. The items included the following: “My supervisor treats employees with respect” and “My supervisor treats employees in a polite manner”.

#### 3.2.5. Control Variable

Considering the control variables used in previous studies analyzing knowledge sharing and creativity, this study used employees’ age, gender, and years of service with their supervisor as control variables.

## 4. Results

Table 1 presents the means, standard deviations, correlations, and reliability. The correlation between variables was consistent with that reported in previous studies. Reliability values are indicated in parentheses at the end of each variable. All have a high value above 0.91.

Table 2 provides the results of the multiple regression analysis conducted to test the hypotheses of this study. Hypotheses 1 and 2 were related to the effect of coworkers’ helping behavior on knowledge sharing and creativity. As suggested by Model 2 in Table 2, coworkers’ helping behavior had a significant effect on knowledge sharing (*r* = 0.18, *p* < 0.01), thus supporting Hypothesis 1, namely that coworkers’ helping behavior has a positive influence on knowledge sharing. Model 4 in Table 2 shows that coworkers’ helping behavior had a significant effect on creativity (*r* = 0.18, *p* < 0.05), supporting Hypothesis 2. The results confirmed that a higher level of coworkers’ helping behavior led to greater knowledge sharing and creativity.

Hypothesis 3 predicts that employees’ knowledge sharing mediates the relationship between coworkers’ helping behavior and employees’ creativity. In addition to supporting Hypotheses 1 and 2, in Models 4 and 5, the effect of coworkers’ helping behavior on employees’ creativity is not significant with the presence of employees’ knowledge sharing (*β* = 0.05, ns in Model 5 vs. *β* = 0.18, *p* < 0.05, in Model 4). The results indicate that employees’ knowledge sharing fully mediates the relationship between coworkers’ helping behavior and employees’ creativity [52]. Furthermore, we performed the bootstrapping method to test the significance of the indirect effect in this study [53]. Bootstrapping 10,000 samples estimated a 95% bias-corrected confidence interval for the indirect effects. As Table 3 shows, the confidence interval does not include zero (ranging from 0.06 to 0.28), indicating that the indirect effect was statistically significant. We further conducted a Sobel test [54] to assess the mediating effect of employees’ knowledge sharing. The results using the normal distribution in Table 3 suggest that the indirect effect of coworkers’ helping behavior on employees’ creativity is significant (*p* = 0.01). Taken together, Hypothesis 3 was supported.

**Figure 2:**
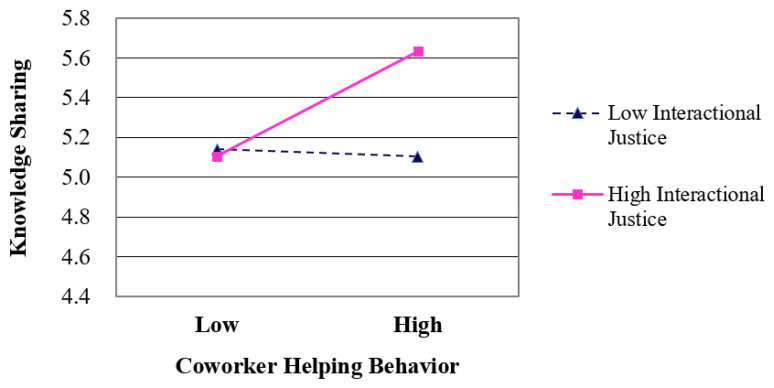
Moderating effect of interactional justice on the relationship between coworkers’ helping behavior and knowledge sharing.

Hypothesis 4 predicts that interactional justice moderates the relationship between coworkers’ helping behavior and employees’ knowledge sharing. Before verifying the moderating effect of interactional justice predicted in Hypothesis 4, centering was performed for the independent and moderating variables with the mean of the variables to reduce multicollinearity between the variables. Model 4 in Table 4 shows that the multiplicative term between coworkers’ helping behavior and interactional justice produced significant results (*r* = 0.16, *p* < 0.05). To more clearly confirm this moderating relationship, the association is presented in Figure 2 in accordance with Aiken and West’s method [55]. As shown in the graph in Figure 2, coworkers’ helping behavior had a stronger positive relationship with knowledge sharing when interactional justice was high than when it was low. Thus, Hypothesis 4 was supported.

Finally, Hypothesis 5 predicted that interactional justice would conditionally influence the strength of the indirect effect of coworkers’ helping behavior on employees’ creativity through employees’ knowledge sharing. We used the SPSS macro developed by Preacher et al. [53] to confirm this moderated mediation. Table 5 shows that the mediation effect is conditional on the level of interactional justice. The indirect effect is stronger (0.24) and significant (confidence interval ranging from 0.12 to 0.40, not crossing zero) for high interactional justice. However, the indirect effect is weaker (−0.02) and not significant for low interactional justice (confidence interval ranging from −0.20 to 0.16, crossing zero).

## 5. Discussion

This study analyzed the relationship between coworkers’ helping behavior, knowledge sharing, and creativity, and demonstrates the moderating effect of the interactional justice of supervisors as a situational variable. The discussions and implications based on the results are as follows.

First, the results of this study confirmed that coworkers’ helping behavior increased employees’ knowledge sharing and creativity. In a rapidly changing global business environment characterized by growing uncertainty for the future, knowledge sharing and creativity are becoming increasingly important for organizations and their employees [6,7]. In this context, encouraging knowledge sharing by promoting cooperation, and helping behavior among employees will serve as a key factor in enhancing competitive advantage. Even from a resource-based perspective, knowledge in an organization is an important internal resource, and the dissemination and sharing of knowledge is essential for preserving and developing that knowledge [8]. Accordingly, this study identified the major antecedent factors of knowledge sharing by revealing that coworkers’ voluntary helping behavior could play a key role in promoting knowledge sharing activities beneficial to their organization.

Second, this study also examined coworkers’ helping behavior as an antecedent variable of creativity. Even though many scholars have proposed the findings of many empirical investigations on the relationship between individual difference and creativity [32,34], coworkers’ helping behavior can have a significant impact on increasing individual creativity. Specifically, voluntarily helping a colleague in difficulty can help the recipient look at the problem from a fresh perspective and increase the possibility of solving the problem in a new and creative way [56]. Thus, the results of this study confirmed that through coworkers’ helping behavior, employees can participate more actively in creative activities that benefit the organization.

Third, this study demonstrated the moderating effect of interactional justice on the relationship between coworkers’ helping behavior and knowledge sharing. A high level of interactional justice means there is a high level of fairness in personal relationships with supervisors and information transmission [26]. Recently, it has been emphasized that interactional justice has a unique feature different from procedural justice [57,58]. Logically, interactional justice is qualitatively different from procedural justice, which focuses on the structural characteristics of procedures [57]. Specifically, no matter how fairly structured a procedure is, there is much room for subjective factors to intervene because interpersonal treatment, which occurs during the implementation of the procedure, can appear differently depending on the decision-maker. Thus, this study suggests that respect, consideration, and fair treatment from supervisors can help employees perceive their organization positively and serve as a catalyst for voluntary helping behavior. The study results showed that coworkers’ helping behavior increased knowledge sharing when interactional justice was high. This study confirmed that interactional justice played an important interactive role in spreading positive outcomes such as knowledge sharing and creativity by maximizing the positive effect of coworkers’ helping behavior to achieve organizational competitiveness.

These findings have the following implications for practice. First, knowledge sharing is an extra-role behavior executed voluntarily for the benefit of other employees, and may be expressed as reciprocation for help received from coworkers. An individual’s willingness to share knowledge with others serves as a key factor for successful knowledge sharing [59]. To do so, companies must build an environment where people can trust each other and share their knowledge while working together. To motivate knowledge sharing, it is important to facilitate regular exchanges among departments, strengthen networking meetings, and use the evaluation or compensation system for knowledge sharing.

Second, understanding and trust in other individuals or departments are greatly needed to promote knowledge sharing and creativity. The mentality of growing together with coworkers for the benefit of the organization as a whole may serve as the foundation of knowledge sharing and creativity. Thus, the management must make an effort to remove the invisible barriers that make it difficult to share knowledge and develop and strengthen a community-like, cooperative organizational culture that unites employees. A good start for knowledge sharing is to provide a forum for mutual communication that involves all supervisors and employees from related departments in resolving pressing corporate issues. Creating an organizational culture where diverse knowledge including know-how gained from failures or mistakes is shared among employees will offer significant value for corporate activities, such as developing new technologies, products, or customer services, thereby increasing work efficiency, reducing cost, and improving corporate productivity.

This study has the following limitations, based on which directions for future research are suggested. First, as the respondents in this study were only from public enterprises, it may be difficult to generalize the results. Future studies should expand the scope of research to cover a wide range of private-sector companies and other organizations. Second, it is difficult to identify causality with the results of this study, which is based on a cross-sectional study design. In other words, the results cannot clearly demonstrate whether coworkers’ helping behavior increases employees’ knowledge sharing and creativity, or vice versa. Therefore, future studies should seek to clarify the causal relationship between variables with a longitudinal and experimental study design.

Third, future studies should examine various factors that hinder knowledge sharing. Knowledge providers may want to keep the knowledge to themselves. For instance, the idea that knowledge is power, perception that sharing knowledge requires much time and effort, and belief that recipients will not fully understand the knowledge they provide may hinder knowledge sharing. In addition, the perception that external knowledge is unacceptable for knowledge recipients or their obsessive attachment to the group to which they belong may make them closed to knowledge from other providers. Moreover, further research is needed to understand whether the organizational culture in which the studies were conducted have an impact on knowledge sharing among coworkers and employees’ creative performance [60]. Additional in-depth research is needed to examine ways to overcome such negative emotions and trigger positive emotions that can maximize knowledge sharing and creativity through helping behavior with the moderating effect of interactional justice.

## 6. Conclusions

Considering that helping behavior among coworkers will play a significant role in promoting knowledge sharing and enhancing creativity among members, these relationships were empirically investigated in this study. The results demonstrated that coworkers’ helping behavior is positively related to employees’ knowledge sharing, which leads to increased levels of employee creativity. Moreover, this study confirmed that the positive and indirect effect of coworkers’ helping behavior on employees’ creativity through employees’ knowledge sharing is stronger when interactional justice is high rather than low. As a result, this study could provide theoretical and practical implications for the importance of coworkers’ helping behavior and supervisors’ interactional justice in promoting knowledge sharing and creativity in order to achieve organizational competitive advantage. Further research on related topics will be not only necessary but also critical to advancing our understanding of this field.

## Figures and Tables

**Table 1:** Descriptive statistics and correlations.

	Variable	Mean	SD	1	2	3	4	5	6	7
1.	Subordinate age	33.47	4.32							
2.	Subordinate gender	1.42	0.49	−0.37 ***						
3.	Tenure with supervisor	12.45	13.34	0.01	0.13 *					
4.	Coworkers’ helping behavior	4.50	0.91	−0.05	−0.01	−0.05	(0.91)			
5.	Interactional justice	4.87	1.12	−0.06	−0.01	0.00	0.25 ***	(0.97)		
6.	Knowledge sharing	5.28	0.94	−0.05	−0.04	−0.09	0.19 **	0.15 *	(0.96)	
7.	Creativity	4.82	1.12	−0.05	−0.11	−0.14 *	0.19 **	0.06	0.73 ***	(0.99)

Note: N = 200. Reliability is given on the diagonal in parentheses. * *p* < 0.05; ** *p* < 0.01; *** *p* < 0.001 (two-tailed).

**Table 2:** Hierarchical regression results for simple mediation.

	Knowledge Sharing			Creativity	
	Model 1	Model 2	Model 3	Model 4	Model 5
Step 1: Control Variables					
Subordinate Age	−0.07	−0.06	−0.10	−0.09	−0.05
Subordinate Gender	−0.06	−0.05	−0.13	−0.13	−0.09
Tenure with Supervisor	−0.08	−0.07	−0.13	−0.12	−0.06
Step 2: Main Effects					
Coworker Helping Behavior		0.18 **		0.18 *	0.05
Step 3: Mediator					
Knowledge Sharing					0.71 ***
Overall F	0.88	2.39	2.60	3.65 **	46.53 ***
R^2^	0.00	0.03	0.02	0.05	0.53
Change in F		6.84 **		6.59 *	202.91 ***
Change in R^2^		0.03		0.03	0.48

Note: N = 200. * *p* < 0.05; ** *p* < 0.01; *** *p* < 0.001 (two-tailed).

**Table 3:** Indirect effect of coworkers’ helping behavior on creativity through employees’ knowledge sharing.

	Indirect Effect and Significance Using Normal Distribution			
Sobel	Effect	SE	Z	*p*
	0.16	0.06	2.57	0.01
	**Bootstrap results for indirect effect**			
Bootstrap	Effect	Boot SE	LL 95% CI	UL 95% CI
	0.16	0.06	0.06	0.28

Note: N = 200. Bootstrap sample size = 10,000. SE = standard error; LL = lower limit; CI = confidence interval; UL = upper limit.

**Table 4:** Results for the moderating effect of interactional justice.

	Knowledge Sharing			
	Model 1	Model 2	Model 3	Model 4
Step 1: Control Variables				
Subordinate Age	−0.07	−0.06	−0.05	−0.06
Subordinate Gender	−0.06	−0.05	−0.05	−0.01
Tenure with Supervisor	−0.08	−0.07	−0.08	−0.10
Step 2: Main Effects				
Coworker Helping		0.18 **	0.16 *	0.13
Step 3: Main Effects				
Interactional Justice			0.11	0.13
Step 4: Moderating Effects				
Coworker Helping × Interactional Justice				0.16 *
Overall F	0.88	2.39	2.39 *	2.87 *
R^2^	0.00	0.03	0.03	0.05
Change in F		6.84 **	2.32	5.05 *
Change in R^2^		0.03	0.01	0.02

Note: N = 200. * *p* < 0.05; ** *p* < 0.01; *** *p* < 0.001 (two-tailed).

**Table 5:** Conditional indirect effects of coworkers’ helping behavior on creativity at values of interactional justice.

		Creativity			
Mediator	Level	Effect	SE	LL 95% CI	UL 95% CI
Knowledge Sharing	Low	−0.02	0.09	−0.20	0.16
	Mean	0.11	0.06	−0.00	0.23
	High	0.24	0.07	0.12	0.40

*Note:* N = 200. Bootstrap sample size = 10,000. LL = lower limit; CI = confidence interval; UL = upper limit.

## References

[1] Reiter-Palmon R., Illies J.J. (2004). Leadership and creativity: Understanding leadership from a creative problem-solving perspective. Leadersh. Q..

[2] Amabile T.M. (1988). A model of creativity and innovation in organizations. Res. Organ. Behav..

[3] Liu D., Gong Y., Zhou J., Huang J.C. (2017). Human resource systems, employee creativity, and firm innovation: The moderating role of firm ownership. Acad. Manag. J..

[4] James K., Drown D., Mumford M.D. (2012). Organizations and creativity: Trends in research, status of education and practice, agenda for the future. Handbook of Organizational Creativity.

[5] Zhou J. (2003). When the presence of creative coworkers is related to creativity: Role of supervisor close monitoring, developmental feedback, and creative personality. J. Appl. Psychol..

[6] Dong Y., Bartol K.M., Zhang Z.X., Li C. (2017). Enhancing employee creativity via individual skill development and team knowledge sharing: Influences of dual-focused transformational leadership. J. Organ. Behav..

[7] Mehmood M.S., Jian Z., Akram U., Akram Z., Tanveer Y. (2021). Entrepreneurial leadership and team creativity: The roles of team psychological safety and knowledge sharing. Pers. Rev..

[8] Jiang Y., Chen C.C. (2018). Integrating knowledge activities for team innovation: Effects of transformational leadership. J. Manag..

[9] Jolly P.M., Kong D.T., Kim K.Y. (2021). Social support at work: An integrative review. J. Organ. Behav..

[10] Lee A., Thomas G., Martin R., Guillaume Y. (2019). Leader-member exchange (LMX) ambivalence and task performance: The cross-domain buffering role of social support. J. Manag..

[11] De Cremer D. (2002). Respect and cooperation in social dilemmas: The importance of feeling included. Personal. Soc. Psychol. Bull..

[12] Hannam K., Narayan A. (2015). Intrinsic motivation, organizational justice, and creativity. Creat. Res. J..

[13] Zhou J., George J.M. (2001). When job dissatisfaction leads to creativity: Encouraging the expression of voice. Acad. Manag. J..

[14] Venkataramani V., Dalal R.S. (2007). Who helps and harms whom? Relational antecedents of interpersonal helping and harming in organizations. J. Appl. Psychol..

[15] Van Dyne L., Cummings L.L., Parks J.M., Staw B.M., Cummings L.L. (1995). Extra-role behaviors: In pursuit of construct and definitional clarity (a bridge over muddied waters). Research in Organizational Behavior: An Annual Series of Analytical Essays and Critical Reviews.

[16] Organ D.W. (1998). Organizational Citizenship Behavior: The Good Soldier Syndrome.

[17] Rhoades L., Eisenberger R. (2002). Perceived organizational support: A review of the literature. J. Appl. Psychol..

[18] Bakker A.B., Demerouti E. (2008). Towards a model of work engagement. Career Dev. Int..

[19] Davenport T.H., Prusak L. (1997). Information Ecology: Mastering the Information and Knowledge Environment.

[20] Hansen M.T. (1999). The search-transfer problem: The role of weak ties in sharing knowledge across organization subunits. Adm. Sci. Q..

[21] Grant R.M. (1996). Toward a knowledge based theory of the firm. Strateg. Manag. J..

[22] Srivastava A., Bartol K.M., Locke E.A. (2006). Empowering leadership in management teams: Effects on knowledge sharing, efficacy, and performance. Acad. Manag. J..

[23] Wang S., Noe R.A. (2010). Knowledge sharing: A review and directions for future research. Hum. Resour. Manag. Rev..

[24] Barney J. (1991). Firm resources and sustained competitive advantage. J. Manag..

[25] Hargadon A.B. (1998). Firms as knowledge brokers: Lessons in pursuing continuous innovation. Calif. Manag. Rev..

[26] Cropanzano R., Prehar C.A., Chen P.Y. (2002). Using social exchange theory to distinguish procedural from interactional justice. Group Organ. Manag..

[27] Watson S., Hewett K. (2006). A multi-theoretical model of knowledge transfer in organizations: Determinants of knowledge contribution and knowledge reuse. J. Manag. Stud..

[28] Smith C.A., Organ D.W., Near J.P. (1983). Organizational citizenship behavior: Its nature and antecedents. J. Appl. Psychol..

[29] Amabile T.M. (1983). The social psychology of creativity: A componential conceptualization. J. Personal. Soc. Psychol..

[30] Amabile T.M. (1997). Motivating creativity in organizations: On doing what you love and loving what you do. Calif. Manag. Rev..

[31] Fürst G., Ghisletta P., Lubart T. (2016). Toward an integrative model of creativity and personality: Theoretical suggestions and preliminary empirical testing. J. Creat. Behav..

[32] Jafri M.H., Dem C., Choden S. (2016). Emotional intelligence and employee creativity: Moderating role of proactive personality and organizational climate. Bus. Perspect. Res..

[33] Puryear J.S., Kettler T., Rinn A.N. (2019). Relating personality and creativity: Considering what and how we measure. J. Creat. Behav..

[34] Zare M., Flinchbaugh C. (2019). Voice, creativity, and big five personality traits: A meta-analysis. Hum. Perform..

[35] Zhou J., Hoever I.J. (2014). Research on workplace creativity: A review and redirection. Annu. Rev. Organ. Psychol. Organ. Behav..

[36] Grodal S., Nelson A.J., Siino R.M. (2015). Help-seeking and help-giving as an organizational routine: Continual engagement in innovative work. Acad. Manag. J..

[37] Gardner H.K., Gino F., Staats B.R. (2012). Dynamically integrating knowledge in teams: Transforming resources into performance. Acad. Manag. J..

[38] Ma Z., Long L., Zhang Y., Zhang J., Lam C.K. (2017). Why do high-performance human resource practices matter for team creativity? The mediating role of collective efficacy and knowledge sharing. Asia Pac. J. Manag..

[39] Hülsheger U.R., Anderson N., Salgado J.F. (2009). Team-level predictors of innovation at work: A comprehensive meta-analysis spanning three decades of research. J. Appl. Psychol..

[40] Nonaka I., Takeuchi H. (1995). The Knowledge Creating Company: How Japanese Companies Create the Dynamics of Innovation.

[41] Colquitt J.A. (2001). On the dimensionality of organizational justice: A construct validation of a measure. J. Appl. Psychol..

[42] Viswesvaran C., Ones D.S. (2002). Examining the construct of organizational justice: A meta-analytic evaluation of relations with work attitudes and behaviors. J. Bus. Ethics.

[43] Colquitt J.A., Conlon D.E., Wesson M.J., Porter C.O., Ng K.Y. (2001). Justice at the millennium: A meta-analytic review of 25 years of organizational justice research. J. Appl. Psychol..

[44] Bies R.J., Moag J.S. (1986). Interactional justice: Communication criteria of fairness. Res. Negot. Organ..

[45] Tyler T.R., Bies R.J., Carroll J. (1990). Beyond formal procedures: The interpersonal context of procedural justice. Applied Social Psychology and Organizational Settings.

[46] Folger R., Cropanzano R. (1998). Organizational Justice and Human Resource Management.

[47] Gurbuz S. (2009). Some possible antecedents of military personnel organizational citizenship behavior. Mil. Psychol..

[48] Blau P.M. (1964). Social Exchange Theory.

[49] Lind E.A., Greenberg J., Cropanzano R. (2001). Fairness heuristic theory: Justice judgements as pivotal cognitions in organizational relations. Advances in Organizational Justice.

[50] Brislin R., Triandis H.C., Berry J.W. (1980). Translation and content analysis of oral and written material. Handbook of Cross-Culture Psychology.

[51] Williams L.J., Anderson S.E. (1991). Job satisfaction and organizational commitment as predictors of organizational citizenship and in-role behaviors. J. Manag..

[52] Baron R.M., Kenny D.A. (1986). The moderator–mediator variable distinction in social psychological research: Conceptual, strategic, and statistical considerations. J. Personal. Soc. Psychol..

[53] Preacher K.J., Rucker D.D., Hayes A.F. (2007). Addressing moderated mediation hypotheses: Theory, methods, and prescriptions. Multivar. Behav. Res..

[54] Sobel M.E. (1982). Asymptotic confidence intervals for indirect effects in structural equation models. Sociol. Methodol..

[55] Aiken L.S., West S. (1991). Multiple Regression: Testing and Interpreting Interactions.

[56] Chiaburu D.S., Harrison D.A. (2008). Do peers make the place? Conceptual synthesis and meta-analysis of coworker effects on perceptions, attitudes, OCBs, and performance. J. Appl. Psychol..

[57] Loi R., Yang J., Diefendorff J.M. (2009). Four-factor justice and daily job satisfaction: A multilevel investigation. J. Appl. Psychol..

[58] Thurston P.W., McNall L. (2010). Justice perceptions of performance appraisal practices. J. Manag. Psychol..

[59] Swift M.L., Virick M. (2013). Perceived support, knowledge tacitness, and provider knowledge sharing. Group Organ. Manag..

[60] Lee A., Legood A., Hughes D., Tian A.W., Newman A., Knight C. (2020). Leadership, creativity, and innovation: A meta-analytic review. Eur. J. Work Organ. Psychol..

